# Positive Impact of Intraoperative Epidural Ropivacaine Infusion on Oncologic Outcomes in Pancreatic Cancer Patients Undergoing Pancreatectomy: A Retrospective Cohort Study

**DOI:** 10.7150/jca.57661

**Published:** 2021-05-27

**Authors:** Wannan Chen, Yaolin Xu, Yueming Zhang, Wenhui Lou, Xiaodan Han

**Affiliations:** 1 Department of Anesthesiology, Zhongshan Hospital Fudan University, 180 Fenglin Road, Shanghai, China.; 2 Department of General Surgery, Zhongshan Hospital Fudan University, 180 Fenglin Road, Shanghai, China.

**Keywords:** pancreatic ductal adenocarcinoma, epidural anesthesia, ropivacaine, overall survival, pancreatectomy

## Abstract

**Background:** Previous literatures have demonstrated that regional anesthesia such as epidural anesthesia may affect long-term survival of cancer patients. In the present study, we conducted a retrospective cohort study to investigate the survival impact of intraoperatively epidural ropivacaine infusion on pancreatic ductal adenocarcinoma (PDAC) patients.

**Methods:** PDAC patients who underwent pancreatic surgery in Zhongshan Hospital Fudan University from January, 2015 to June, 2018 were included. The surgical procedure was performed under combined endotracheal general anesthesia and thoracic epidural anesthesia, and patient-controlled epidural analgesia (PCEA) with 0.12% ropivacaine was given after surgery for further pain control. Patients were divided into two groups according to their intraoperative epidural ropivacaine concentration: high (0.375%-0.5%) and low (0.15%-0.25%). Survival outcome was compared between groups.

**Results:** A total of 215 patients were enrolled and their baseline characteristics were balanced between groups, except that patients with high concentration ropivacaine received higher total dose opioid and had longer operative time. Resected PDAC patients who were administrated with high concentration ropivacaine through epidural catheter intraoperatively had improved overall survival (median overall survival, mOS, high VS low, 37.6 VS 23.7 months, p=0.04). High epidural ropivacaine concentration was an independent prognostic factor (hazard ratio [HR]=0.65, 95% confidence interval [CI], 0.44-0.94; p=0.03). Subgroups analyses shown that T3M0 PDAC patients with preoperative CA 19-9 higher than 200 U/ml, negative resection margin, and those without tumor deposit and adjuvant radiotherapy could benefit from high concentration of ropivacaine.

**Conclusion:** Intraoperatively epidural infusion with high concentration of ropivacaine was associated with improved OS in PDAC patients undergoing pancreatectomy.

## Introduction

Pancreatic ductal adenocarcinoma (PDAC) is high malignant tumor with dismal prognosis, causing approximately 85,000 and 40,000 death per year in China and the United States 1. Without obvious early symptoms, majority of patients are diagnosed in advanced stage and lost chance of curative surgery. As a result, the five-year survival rate remains at only 9% 2.

Currently, surgical resection is only possible curative treatment for pancreatic cancer. However, surgical intervention might impair cell-mediated immunity and activated circulating tumor cells implantation which leave patients susceptible to develop of postoperative recurrence or metastasis 3,4. Multiple retrospective studies have shown that regional anesthesia improved patient outcome after surgery in bladder, breast and lung cancers 5-7. In addition, regional anesthesia such as epidural anesthesia may blunt the pro-metastatic effect of surgical stress and inhibit tumor growth and metastasis, especially in high concentrations 8. Tyler et al. also observed that perioperative epidural analgesia was a positive prognostic factor in patients undergoing resection of pancreatic cancer 9.

Ropivacaine was a frequently-used long-acting amide regional anaesthetic agent. Baptista-Hon demonstrated that ropivacaine decreases the ability of cell invasion in colon cancer by regulating NaV1.5 channel activity 10. Meanwhile, ropivacaine could inhibit lung and breast cancer cell proliferation by arresting cell cycle *in vitro* 6,7. However, it is unclear whether regional anesthesia with ropivacaine during pancreatic surgery was associated with any improvement in oncologic outcomes. Thus, we conducted this retrospective study to investigate whether there is association between intraoperative epidural ropivacaine concentration and long-term clinical outcome of PDAC patients.

## Materials and Methods

### Study Population

**Figure 1:**
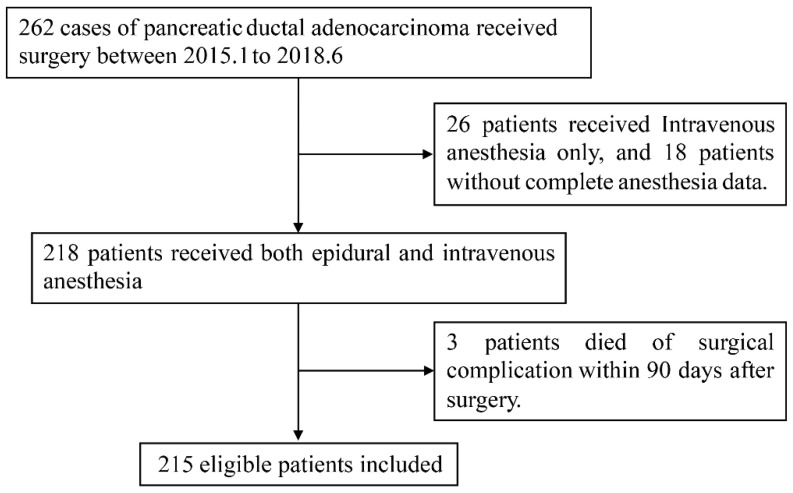
Flowchart of patient selection.

We enrolled PDAC patients who received curative resection of primary pancreatic cancer in our institution from January, 2015 to June, 2018. All the enrolled patients were selected based on the following criteria: 1) aged 18 years or older, 2) with pathologically confirmed evidence of PDAC, 3) without history of other primary malignant tumor, 4) no history of antitumor treatment before surgery, 5) received combined general-epidural anesthesia, 6) with complete clinicopathologic data. Patients who died within 90 days after surgery because of postoperative complications were not able to evaluate their long-term oncologic outcome and thus were excluded from the study. A flowchart of patient selection was shown in Figure 1.

### Study Design

In the present study, we aimed to evaluate whether intraoperative epidural ropivacaine infusion was associated with long-term oncology outcome of PDAC patients. The primary endpoint was overall survival (OS). OS was defined as the period from the date of surgery to the date of death or the last follow-up. The secondary endpoint was disease free survival (DFS), which was defined as time from surgery to either local recurrence or distant metastasis occurred. The latest follow-up was performed in October, 2020. The survival data was obtained from medical records and follow-up telephone interviews.

This study was approved by the Ethics Board of Zhongshan Hospital, Fudan University (B2020-409R). Informed consent was waived because the data was anonymized before analysis.

### Clinicopathologic Data

Electronic medical records of recruited patients, including demographic characteristics, pathologic results, preoperative serum parameters, and the American Society of Anesthesiologists (ASA) physical status classification, were collected from the database of Zhongshan Hospital, Fudan University clinical information system. The data of American Joint Committee on Cancer (AJCC) 8^th^ TNM stage, tumor differentiation, microvascular invasion (MVI), peripancreatic fat invasion (FI), neural invasion (NI), surgical margin, and tumor deposit were extracted from final pathologic reports. A positive resection margin was defined as either microscopic or macroscopic presence of tumor cells at definite resection margins. Tumor deposit was a microscopic or macroscopic tumor nodule located in the peripancreatic fat or lymphatic drainage bed of pancreatic cancer. A carbohydrate antigen 19-9 (CA 19-9) cut point of 35 U/ml was set to dichotomize patients with normal and elevated values based on a research of Aldakkak and colleagues. Patients with an elevated CA 19-9 level were then further stratified into low (36-200) and high (>200) groups 11.

### Exposure Variable

The aim of the study was to observe the effect of intraoperative epidural ropivacaine concentration on clinical outcomes after pancreatic surgery. Patients in low concentration group received 0.15%-0.25% ropivacaine through the epidural catheter intraoperatively, while those who received 0.375%-0.5% ropivacaine were belonged to high concentration group.

The anesthesia manipulation was conducted according to the attending anesthesiologist's discretion. Since high concentration of epidurally administrated ropivacaine may cause a significant hemodynamic change^12^,^13^, anesthesiologists in our medical center prefer to delivered lower concentration of ropivacaine in elder patients for more stable hemodynamic and to reduce potential perioperative cardiovascular complications Meanwhile, higher epidural ropivacaine concentration provided not only regional analgesia but also a better relaxation of abdominal muscles 14-16. Thus, anesthesiologists in our medical center prefer to administrate high dose of ropivacaine in patients who received radical, complicated and time-consuming surgeries, such as pancreaticoduodenectomy in the present study.

### Anesthesia Care

All the intraoperative care was provided by the consultant anesthesiologists. Upon entering the operating room, patients were received epidural puncture at the mid-thoracic level (T8-T10). The procedures were done under endotracheal general anesthesia with epidural anesthesia. General anesthesia was induced with sufentanil (0.3-0.5 µg/kg), propofol (target-controlled infusion, effect-site concentration: 3.0-4.0 µg/kg), and rocuronium (0.6 mg/kg). Anesthesia was maintained with desflurane (0.8MAC) in an oxygen/air mixture. Ventilator was set as follows: tidal volume 8 ml/kg, respiratory rate 10-12/min, keeping EtCO2 between 35-45 mmHg. Either sufentanil or fentanyl and rocuronium were given repeatedly as necessary throughout the surgery. For epidural anesthesia, all patients received a stress volume (8-10ml) of 0.1875-0.5% ropivacaine before the incision and a bolus volume (4-5 ml) each hour. Patients were sent to post anesthesia care unit (PACU) for a further one-hour monitored care. Per the empirical therapy in our center, all patients received a patient-controlled epidural analgesia (PCEA) pump (0.12% ropivacaine and 0.4 µg/ml sufentanil, background: 3 ml/hour, bolus 4 ml/hour, lockout time: 10 min) to control the postoperative pain. The dose conversion ratio for fentanyl/sufentanil was 6 based on previous research and the total opioid dose was defined as intraoperative opioid dose and calculated as relative total fentanyl administration 17.

### Statistical Analysis

Data analyses were conducted and graphics were generated by using R project 4.04 (heep://www.r-project.org/) for Windows and IBM SPSS Statistics 22.0 version. Categorical variables were reported as frequencies and percentages, while continuous variables were described as medians. Normality and homogeneity of variance were tested using Shapiro-Wilk test and Levene's test. T-tests or Mann-Whitney U tests were used to compare continuous variables and Fisher's exact test or Pearson's chi-squared test was used to analyze categorical variables between groups. The Cox proportional hazards model was used to estimate the hazard ratio (HR) of death. The significant statistical variables (p<0.05) in univariate Cox regression analysis were included into multivariate analysis using a forward conditional method to identify the independent prognostic factors for survival. Kaplan-Meier (KM) method was used to calculate OS, DFS, and follow-up period. Exploratory subgroup analyses were conducted to investigate the potential effect of epidural ropivacaine infusion on oncologic outcomes in certain subgroups and were illustrated by forest plot.

## Results

### Study population characteristics

A total of 215 PDAC patients were included in the present study and the clinicopathologic characteristics were summarized in Table 1. The proportion of patients with higher and lower concentration of ropivacaine infusion was 55.3% (n=119) and 44.7% (n=96), respectively. None of the baseline characteristics differed significantly between two groups except for total opioid dose (high VS low, 198.9 VS 180 μg fentanyl, p=0.049) and operation duration (high VS low, 239.3 VS 187.1 min, p<0.001). Meanwhile, we found that younger male patients who accepted pancreatoduodenectomy were more likely to receive higher dose of epidural ropivacaine infusion intraoperatively (high VS low; male, 63.9% VS 51%, p=0.071; mean age, 62.7 VS 64, p=0.21; pancreatic head lesion, 59.7% VS 47.9%, p=0.083), although the difference were insignificant.

### Epidural anesthesia with higher concentration of ropivacaine was associated with better OS in PDAC patients

**Figure 2:**
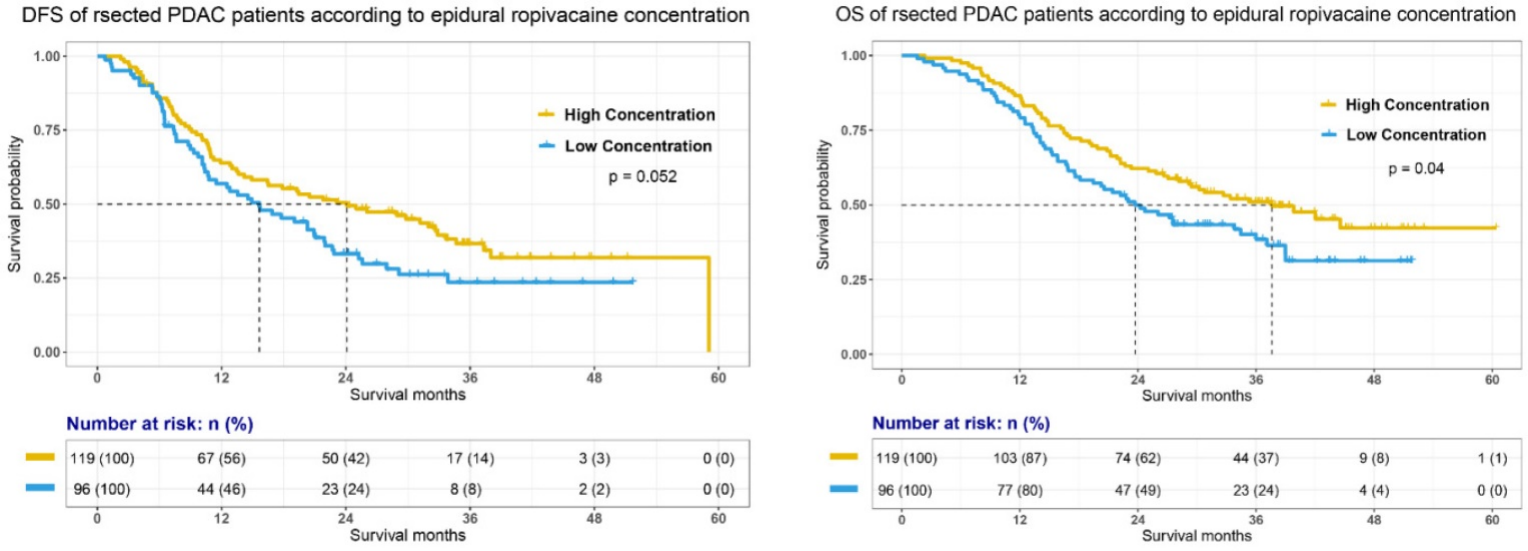
Disease free survival (DFS) and overall survival (OS) of pancreatic ductal adenocarcinoma (PDAC) patients undergoing pancreatectomy according to the epidural ropivacaine concentration they received.

In this study, median follow-up time for whole cohort was 38.3 months (95% confidence interval, 95% CI, 37.2 to 39.7). Survival analysis (Fig. 2) demonstrated that high dose intraoperative epidural ropivacaine infusion was associated with improved OS in PDAC patients (median OS, mOS, high VS low, 37.6 VS 23.7 months, p=0.04). Although it was statistically insignificant, we also observed a better DFS in high concentration group (median DFS, mDFS, high VS low, 24.1 VS 15.6 months, p=0.052). Cox proportional hazards models was constructed to assess association of relevant variables with OS and was summarized in Table 2. Univariate Cox regression analysis indicated that T stage (p<0.01), N stage (p<0.01), M stage (p=0.019), adjuvant chemotherapy (p=0.04), preoperative CA 19-9 level (p<0.01), NI (p<0.01), FI (p<0.01), tumor deposits (p<0.01), tumor differentiation (p<0.01), and epidural ropivacaine concentration (p=0.041) were all significantly associated with OS. Further multivariate Cox regression analysis confirmed T stage (p<0.01), N stage (p<0.01), M stage (p<0.01), adjuvant chemotherapy (p=0.04), preoperative CA 19-9 level (p<0.01), tumor differentiation (p=0.042), and epidural ropivacaine concentration (p=0.03) as independent prognostic factors and higher dose of intraoperative epidural ropivacaine infusion remained as a significant protective factor in the model (HR=0.65, 95% CI, 0.44-0.94; p=0.03).

### Exploratory subgroup analyses

**Figure 3:**
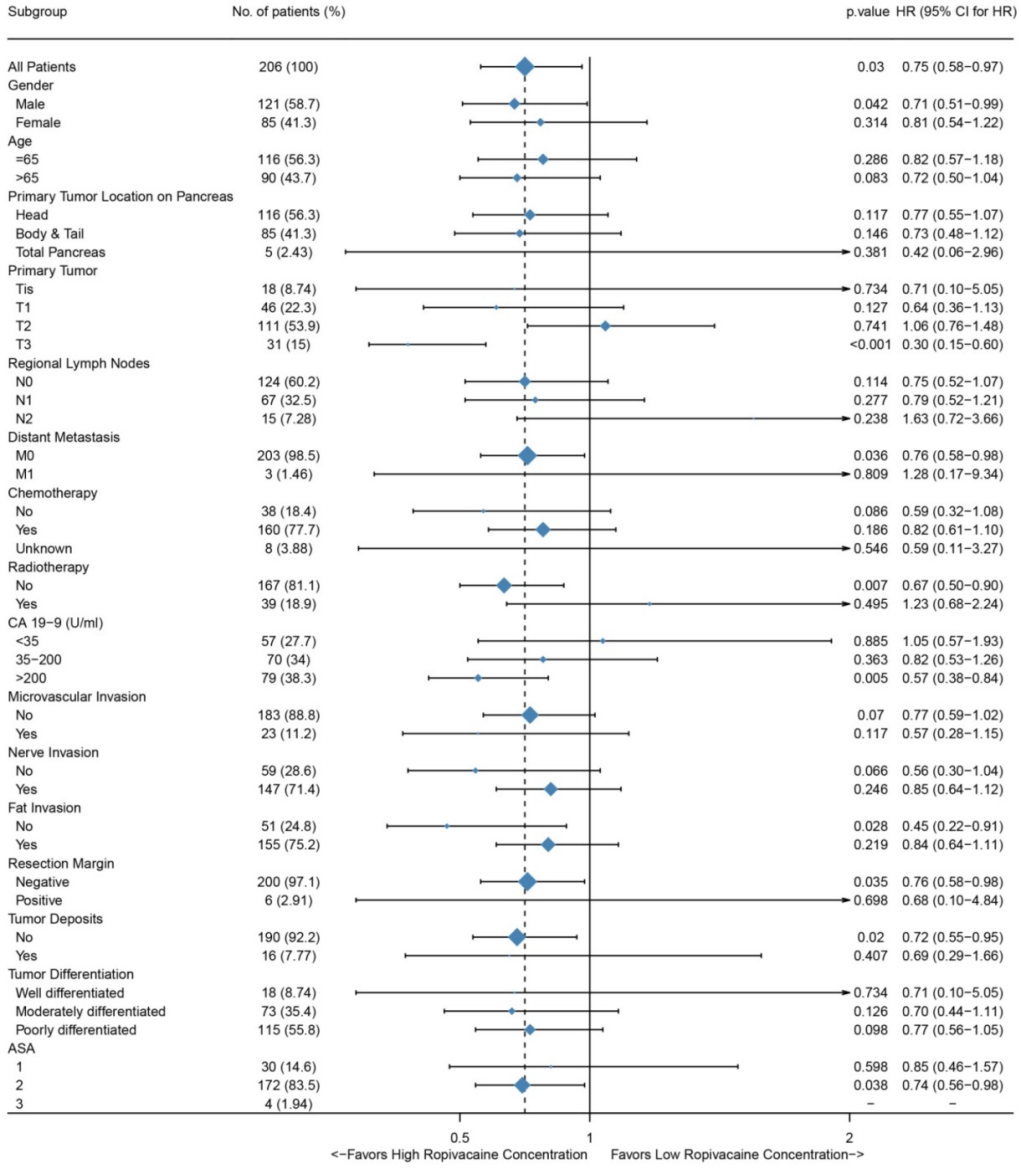
Forest plots of hazard ratios (HR) according to subgroup analysis for OS. The x-axis shows the HR and 95% confidence interval (CI) of each subgroup. The size of the boxes represents the relative number of patients in each subgroup.

To further investigate whether epidural ropivacaine concentration was a prognostic factor in certain patient subgroups, we performed exploratory subgroup analyses. As shown in Figure 3, the forest plot demonstrated that high ropivacaine concentration had better prognostic potential in male (HR=0.71, 95%CI: 0.51-0.99, p=0.042) patients with ASA II (HR=0.74, 95%CI: 0.56-0.98, p=0.038). PDAC patients with T3 stage disease (HR=0.3, 95%CI: 0.15-0.6, p<0.001) and preoperative CA 19-9 higher than 200 U/ml (HR=0.57, 95%CI: 0.38-0.84, p=0.005) were more likely to benefit from higher dose ropivacaine. Meanwhile, high ropivacaine concentration remained as a positive prognostic factor in patients with negative resection margin (HR=0.76, 95%CI: 0.58-0.98, p=0.035), and without distant metastasis (HR=0.76, 95%CI: 0.58-0.98, p=0.036), FI (HR=0.45, 95%CI: 0.22-0.91, p=0.028), tumor deposit (HR=0.72, 95%CI: 0.55-0.95, p=0.02), and adjuvant radiotherapy (HR=0.67, 95%CI: 0.5-0.9, p=0.007).

**Figure 4:**
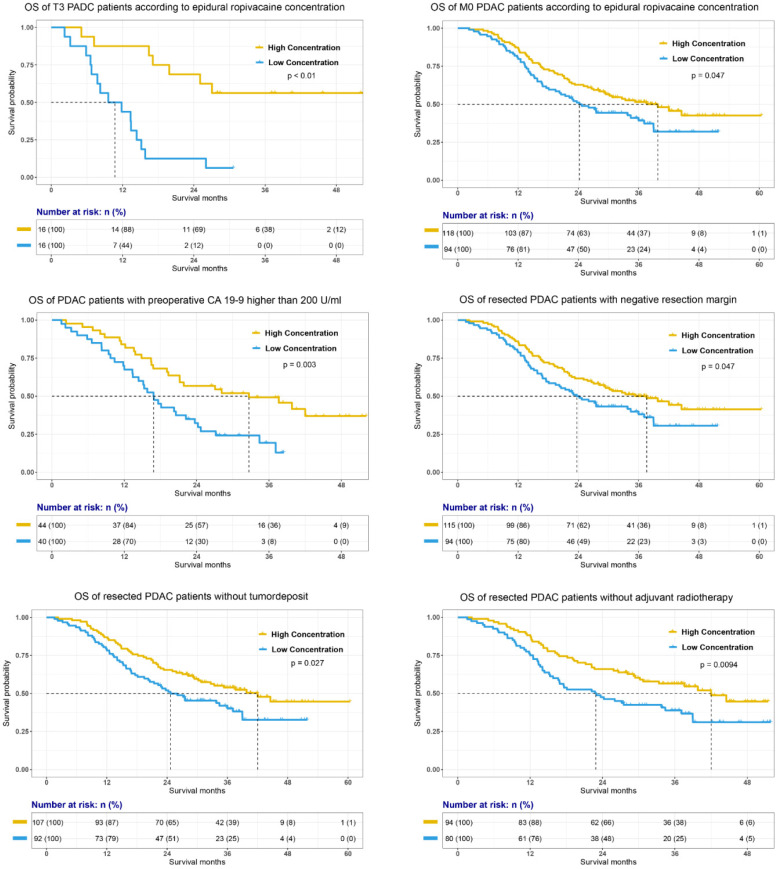
Survival analyses of specific subgroups according to the epidural ropivacaine concentration.

The prognostic value of epidural ropivacaine concentration in these subgroups was further confirmed by the survival analyses showed in Figure 4 and supplemental Figure 1. The results indicated the possible prognostic effect of epidural ropivacaine concentration in PDAC patients with T3M0 disease, preoperative CA19-9 higher than 200 U/ml, negative resection margin, and those without tumor deposit and adjuvant radiotherapy.

## Discussion

In the present study, we investigated the association of intraoperative epidural administration of ropivacaine and survival outcome of PDAC patients who received curative surgery. Survival analyses showed that patients who received 0.375%-0.5% ropivacaine through the epidural catheter intraoperatively had better OS. Meanwhile, high epidural ropivacaine concentration was an independent protective factor in Cox proportional hazards model. Further subgroup analyses demonstrated the possible prognostic effect of epidural ropivacaine concentration in T3M0 PDAC patients with preoperative CA 19-9 higher than 200 U/ml, negative resection margin, and those without tumor deposit and adjuvant radiotherapy.

In recent years, the effect of intraoperative anesthetic management on long-term clinical outcome of cancer patients has become an interesting research topic. Several retrospective studies reported that local anesthesia, especially perioperative epidural analgesia, was related to better survival and a lower incidence of local recurrence or distant metastasis in cancer patients 5-7,18-21. Tyler et al. observed the association between use of epidural anesthesia during primary pancreatic cancer resection and improved survival among PDAC patients 9. This was consistent with our present study which demonstrated that PDAC patients who intraoperatively received 0.375%-0.5% ropivacaine through epidural catheter had better OS.

There is not standard institutional protocol for ropivacaine dose selection in our medical center. However, as mentioned above, we prefer to deliver higher dose of ropivacaine for younger patients receiving radical and time-consuming surgeries to reduce potential perioperative complications and to provide better abdominal muscles relaxation. These were consistent with our analyses in Table 1. Patients in high ropivacaine concentration group had longer operation duration. Also, patients receiving higher dose of ropivacaine seemed to be younger and had higher proportion of pancreatic head tumor, and thus had more pancreaticoduodenectomy procedures. Again, more complicated surgical procedures and longer operation time could also explain increased dosage of opioid in high ropivacaine concentration group.

Interestingly, we found that PDAC patients with T3M0 disease and preoperative CA 19-9 level higher than 200 U/ml, negative resection margin, and those without tumor deposit and adjuvant radiotherapy were more likely to benefit from high concentration of ropivacaine. As shown in Table 2, both T3 and preoperative CA 19-9 higher than 200 U/ml were negative prognostic factors for PDAC patients (T3, HR=1.57, 95%CI: 1.44-1.72, p<0.01; CA 19-9 > 200 U/ml, HR=1.76, 95%CI: 1.04-2.99, p=0.035). With manageable side effects of analgesics, further clinical research was needed to investigate whether higher epidural ropivacaine concentration could provide survival benefits for PDAC patients, especially for those with preoperative CA 19-9 higher than 200 U/ml, and those whose preoperative imaging assessment showed that primary pancreatic tumor was >4 cm in greatest dimension without distant metastasis.

In Table 1, we found that PDAC patients in 0.375%-0.5% ropivacaine group received increased opioid dosing and Cox proportional model demonstrated that total opioid dose was not a prognostic factor. However, previous literatures showed that reduced opioid dosing would potentially improve perioperative immune function and postoperative long-term survival 22,23. Thus, difference in opioid dose may not account for survival benefit of high dose ropivacaine.

Previous literatures have demonstrated that local anesthetics exerted cytotoxic effect on neural cells in a concentration-dependent fashion 24,25. Meanwhile, ropivacaine was proven to inhibit lung and breast cancer cell proliferation by inducing cell cycle arrest *in vitro* 6,7. In addition, local-anesthetic-sensitive voltage-gated ion channels functionally expressed in a variety of carcinomas and also lymphocytes, and have shown to enhance metastatic cell behaviors 26-30. Accumulating evidences have demonstrated that ropivacaine could block voltage-gated ion channels activity, and thus suppress invasiveness of cancer cells and directly affect the local immune response to surgery 10,26,31. Meanwhile, we found the trend that patients in high concentration group seemed to achieve better DFS in the present study. Thus, the unexpected survival benefit of high concentration ropivacaine may be due to its inhibitory effect on cancer cell proliferation and migration. However, these effects of ropivacaine were shown only *in vitro*. The translation of these effects into clinical outcomes remained unclear.

Several important limitations are inherent in this study. First, this was a retrospective study associating with bias and confounding owing to unmeasured variables that might influence the survival outcome. Second, we did not include preoperative nutritional status and details of adjuvant therapy (e.g. chemotherapy regimen and duration of chemotherapy) in the study. However, these variables might have a significant impact on primary endpoint. Third, our results relate to only ropivacaine epidural anesthesia and do not translate to other neuraxial anesthetic techniques. The association between anesthetic techniques and oncologic outcomes among patients with pancreatic cancer need to be better characterized from a pathophysiological standpoint.

In conclusion, resected PDAC patients who received high concentration of ropivacaine through the epidural catheter intraoperatively had improved OS. Further prospective study is needed to investigate the impact of local anesthesia on oncology outcome of PDAC patients who received primary tumor resection.

## Supplementary Material

Supplementary figure.

## Figures and Tables

**Table 1:** Characteristic of pancreatic cancer patients at baseline

	Epidural Ropivacaine Concentration			
	Total (n=215)	High (n=119)	Low (n=96)	p-value
**Gender**				
Male	125 (58.1%)	76 (63.9%)	49 (51.0%)	0.071#
Female	90 (41.9%)	43 (36.1%)	47 (49.0%)	
**Age**				
Mean (SD)	63.3 (±8.6)	62.7 (±8.5)	64.0 (±8.9)	0.21*
**Primary tumor location on pancreas**				
Head	117 (54.4%)	71 (59.7%)	46 (47.9%)	0.083#
Body & Tail	93 (43.3%)	44 (37.0%)	49 (51.0%)	
Total Pancreas	5 (2.3%)	4 (3.4%)	1 (1.0%)	
**AJCC 8th stage**				
Carcinoma *in situ*	19 (8.8%)	11 (9.2%)	8 (8.3%)	0.4#
IA	32 (14.9%)	23 (19.3%)	9 (9.4%)	
IB	60 (27.9%)	34 (28.6%)	26 (27.1%)	
IIA	16 (7.4%)	9 (7.6%)	7 (7.3%)	
IIB	70 (32.6%)	34 (28.5%)	36 (37.5%)	
III	15 (7.0%)	7 (5.9%)	8 (8.3%)	
IV	3 (1.4%)	1 (0.8%)	2 (2.1%)	
**Primary tumor**				
Tis	19 (8.8%)	11 (9.2%)	8 (8.3%)	0.29#
T1	46 (21.4%)	31 (26.1%)	15 (15.6%)	
T2	117 (54.4%)	60 (50.4%)	57 (59.4%)	
T3	32 (14.9%)	16 (13.4%)	16 (16.7%)	
T4	1 (0.5%)	1 (0.8%)	0 (0.0%)	
**Regional Lymph Nodes**				
N0	126 (59.2%)	77 (65.3%)	49 (51.6%)	0.13#
N1	72 (33.8%)	34 (28.8%)	38 (40.0%)	
N2	15 (7.0%)	7 (5.9%)	8 (8.4%)	
**Distant Metastasis**				
M0	212 (98.6%)	118 (99.2%)	94 (97.9%)	0.59#
M1	3 (1.4%)	1 (0.8%)	2 (2.1%)	
**Chemotherapy**				
No	39 (18.1%)	20 (16.8%)	19 (19.8%)	0.77#
Yes	166 (77.2%)	94 (79.0%)	72 (75.0%)	
Unknown	10 (4.7%)	5 (4.2%)	5 (5.2%)	
**Radiotherapy**				
No	174 (80.9%)	94 (79.0%)	80 (83.3%)	0.29#
Yes	40 (18.6%)	25 (21.0%)	15 (15.6%)	
Unknown	1 (0.5%)	0 (0.0%)	1 (1.0%)	
**CA 19-9 (U/ml)**				
<35	59 (27.4%)	32 (26.9%)	27 (28.1%)	0.64#
35-200	72 (33.5%)	43 (36.1%)	29 (30.2%)	
>200	84 (39.1%)	44 (37.0%)	40 (41.7%)	
**Microvascular Invasion**				
No	189 (87.9%)	105 (88.2%)	84 (87.5%)	1#
Yes	26 (12.1%)	14 (11.8%)	12 (12.5%)	
**Nerve Invasion**				
No	63 (29.3%)	36 (30.3%)	27 (28.1%)	0.76#
Yes	152 (70.7%)	83 (69.7%)	69 (71.9%)	
**Fat Invasion**				
No	54 (25.1%)	31 (26.1%)	23 (24.0%)	0.75#
Yes	161 (74.9%)	88 (73.9%)	73 (76.0%)	
**Resection Margin**				
Negative	209 (97.2%)	115 (96.6%)	94 (97.9%)	0.69#
Positive	6 (2.8%)	4 (3.4%)	2 (2.1%)	
**Tumor Deposits**				
No	199 (92.6%)	107 (89.9%)	92 (95.8%)	0.12#
Yes	16 (7.4%)	12 (10.1%)	4 (4.2%)	
**Tumor Differentiation**				
Well differentiated	19 (8.8%)	11 (9.2%)	8 (8.3%)	0.37#
Moderately differentiated	78 (35.9%)	39 (32.8%)	39 (40.6%)	
Poorly differentiated	118 (54.9%)	69 (58.0%)	49 (51.0%)	
**Total Opioid Dose (Fentanyl, μg)**				
Mean (SD)	190.5 (±71.1)	180.0 (±67.3)	198.9 (±73.3)	0.049*
**ASA score**				
1	31 (14.4%)	15 (12.6%)	16 (16.7%)	0.73#
2	180 (83.7%)	101 (84.9%)	79 (82.2%)	
3	4 (1.9%)	3 (2.5%)	1 (1.0%)	
**Surgical Procedure**				
Open surgery	215 (100.0%)	96 (100.0%)	119 (100.0%)	1
**Clavien-Dindo Classification**				
<3	211 (98.1%)	95 (99.0%)	116 (97.5%)	0.63#
≥3	4 (1.9%)	1 (1.0%)	3 (2.5%)	
**Operation Duration (min)**				
Mean (SD)	216.0 (±107.6)	187.1 (±88.3)	239.3 (±116.2)	< 0.001*

#, Mann-Whitney U test; *, independent sample t-test. SD, standard deviation; AJCC, American Joint Committee on Cancer; CA 19-9, carbohydrate antigen 19-9; ASA, American Society of Anesthesiologists.

**Table 2:** Cox regression analysis for overall survival

Variables	Univariate		Multivariate	
	HR	p-value	HR	p-value
**Gender**				
Male	1 (as reference)	0.803		
Female	0.955 (0.664-1.37)			
**Age**				
Mean (SD)	1.02 (0.9982-1.042)	0.073		
**Primary tumor location on pancreas**				
Head	1 (as reference)	0.58		
Body & Tail	0.884 (0.613-1.27)	0.509		
Others	0.561 (0.138-2.29)	0.421		
**Primary Tumor**				
Tis	1 (as reference)	<0.01	1 (as reference)	<0.01
T1	6.75 (1.6-28.5)	<0.01	1.05 (1.02-1.96)	<0.01
T2	9.02 (2.21-36.8)	<0.01	1.36 (1.21-1.52)	<0.01
T3	13 (3.06-55.5)	<0.01	1.62 (1.44-1.81)	<0.01
**Regional Lymph Nodes**				
N0	1 (as reference)	<0.01	1 (as reference)	<0.01
N1	1.63 (1.11-2.38)	0.012	1.48 (1.24-1.69)	<0.01
N2	2.92 (1.56-5.45)	<0.01	1.72 (1.41-1.99)	<0.01
**Distant Metastasis**				
M0	1 (as reference)	0.019	1 (as reference)	<0.01
M1	5.79 (1.82-18.4)		3.72 (2.71-5.28)	
**Chemotherapy**				
No	1 (as reference)	0.04	1 (as reference)	<0.01
Yes	0.672 (0.429-0.924)		0.35 (0.21-0.59)	
**Radiotherapy**				
No	1 (as reference)	0.436		
Yes	1.19 (0.77-1.84)			
**CA 19-9 (U/ml)**				
<35	1 (as reference)	<0.01	1 (as reference)	<0.01
35-200	1.74 (1.04-2.91)	0.036	1.27 (0.74-2.28)	0.322
>200	2.42 (1.48-3.95)	<0.01	1.76 (1.04-2.99)	0.031
**Microvascular Invasion**				
No	1 (as reference)	0.313		
Yes	1.3 (0.79-2.15)			
**Nerve Invasion**				
No	1 (as reference)	<0.01	1 (as reference)	0.28
Yes	2.47 (1.54-3.96)		1.33 (0.78-2.28)	
**Fat Invasion**				
No	1 (as reference)	<0.01	1 (as reference)	0.08
Yes	2.78 (1.66-4.64)		1.61 (0.941-2.98)	
**Resection Margin**				
Negative	1 (as reference)	0.252		
Positive	0.485 (0.12-1.96)			
**Tumor Deposits**				
No	1 (as reference)	<0.01	1 (as reference)	0.289
Yes	2.37 (1.32-4.23)		1.44 (0.73-2.92)	
**Tumor Differentiation**				
Well differentiated	1 (as reference)	<0.01	1 (as reference)	0.042
Moderately differentiated	6.52 (1.57-27.1)	<0.01	2 (0.12-34.1)	0.616
Poorly differentiated	10.8 (2.65-44)	<0.01	2.55 (0.122-46.9)	0.45
**ASA**				
1	1 (as reference)	0.078		
2	0.748 (0.466-1.2)	0.229		
3	0.275 (0.037-2.05)	0.208		
**Total Opioid Dose (Fentanyl, μg)**				
Mean (SD)	0.998 (0.994-1.004)	0.138		
**Clavien-Dindo Classification**				
<3	1 (as reference)	0.836		
≥3	0.863 (0.213-3.49)			
**Epidural Ropivacaine Concentration**				
Low	1 (as reference)	0.03	1 (as reference)	0.03
High	0.75 (0.58-0.97)		0.65 (0.44-0.94)	

HR, hazard ratio; SD, standard deviation; AJCC, American Joint Committee on Cancer; CA 19-9, carbohydrate antigen 19-9; ASA, American Society of Anesthesiologists.
