# Supplementary material for: Positive Impact of Intraoperative Epidural Ropivacaine Infusion on Oncologic Outcomes in Pancreatic Cancer Patients Undergoing Pancreatectomy: A Retrospective Cohort Study
Source: J Cancer. 2021 May 27;12(15):4513–21. doi: 10.7150/jca.57661 (PMC8210573; doi:10.7150/jca.57661)
Supplement: Supplementary file 1 — Supplementary figure. [file jcav12p4513s1.pdf]

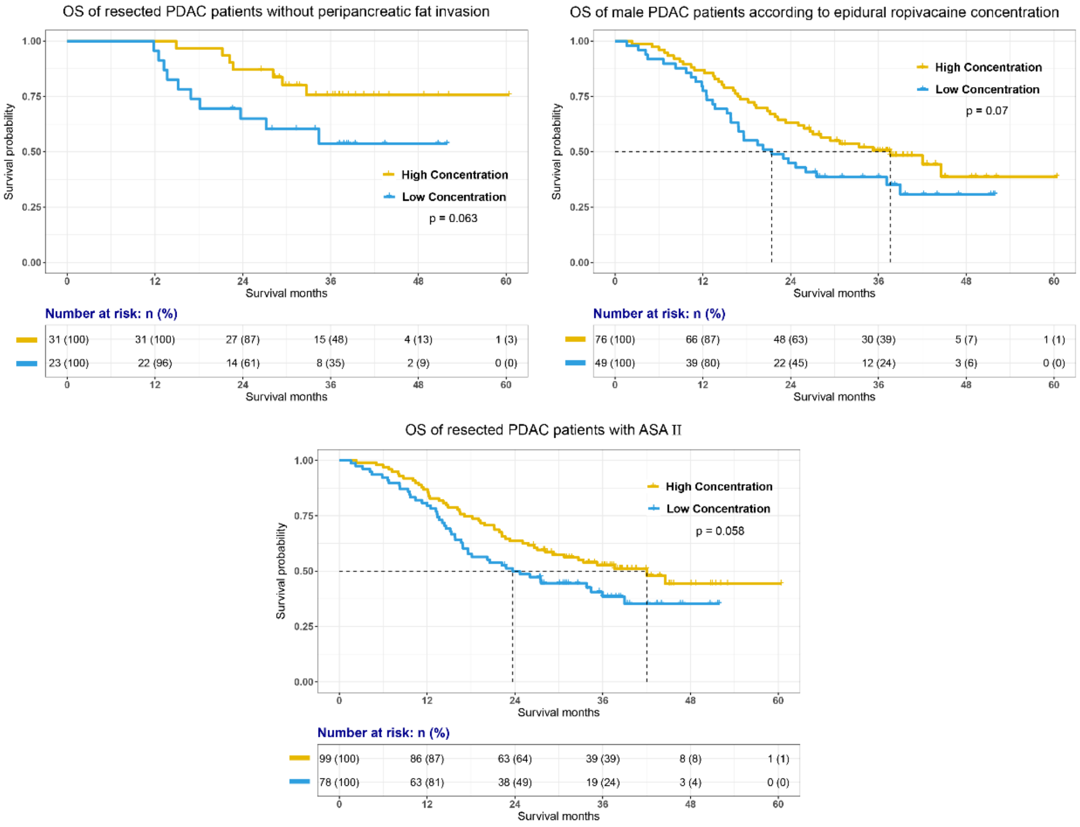

Figure S1. Survival analyses of specific subgroups according to the epidural ropivacaine concentration.
